# Decoupled *in-plane* Dipole Resonance Modulated Colorimetric Assay-Based Optical Ruler for Ultra-Trace Gold (Au) Detection

**DOI:** 10.1038/s41598-018-19148-w

**Published:** 2018-01-17

**Authors:** Ajoy Mandal, Maireyee Bhattacharya, Denis V. Kuznetsov, Tapas Ghosh, Sudeshna Das Chakraborty, Biswarup Satpati, Vsevolod Mazov, Dulal Senapati

**Affiliations:** 1 Chemical Sciences Division, Saha Institute of Nuclear Physics, HBNI, 1/AF Bidhannagar, Kolkata, 700064 India; 2 Surface Physics & Material Science Division, Saha Institute of Nuclear Physics, HBNI, 1/AF Bidhannagar, Kolkata, 700064 India; 3 Department of Functional Nanosystems and High Temperature Materials, National University of Science and Technology ‘MISiS’, Leninsky, Prospect 4, 119049 Moscow, Russia

## Abstract

Decoupling of different plasmon resonance modes (*in-plane*, and *out-of-plane* dipole and quadrupole resonances) by tuning nanoparticle’s size and shape offers a new field of plasmonics as colorimetric assay-based optical-ruler for ultra-trace sensing. Driven by its low cost, easy to perform and efficient way to measure trace level (up to 30 *ppt* in presence of common mining elements in natural gold ore) abundance, this study develops a highly selective and ultrasensitive turn-on colorimetric sensor to detect gold-ion from environmental samples. Different level of gold-ion tracer makes size variable spherical- and disc-shaped silver nanoparticles when added to a ‘growth solution’ which results decoupling of *in-plane* dipole resonance from *in-plane* quadrupole and *out-of-plane* dipole resonances with a wide range of *in-plane* dipole plasmon tunability to generate different colors. This color-coded sensing of gold-ion shows high selectivity and ultrasensitivity over other metal ions in the *ppt* level with an impurity aberration limit of 1 *ppm*. A plausible explanation explains the possible role of catalytic gold-ion to initiate unfavorable silver ion (Ag^+^) reduction by ascorbic acid to generate silver nanoparticles. Proposed technology has been applied in real mining sample (Bugunda Gold Deposit, Tajikistan) to detect gold concentration from ores to find potential application in mining technology.

## Introduction

Development of reliable techniques with greater specificity and ultra-sensitivity has attracted a lot more interest in the field of sensor technology whether that could be an electrical or optical signal output. Avoiding the intense debate, it is well accepted that the optical sensors are easier to perform as well as less complicated to understand and hence more popular for societal cause. Out of several optical sensors, colorimetric sensor is the cheapest one due to the efficient readout by our own eyes as the device and does not seek an extra detector to add up additional cost of sensing. In the past, Chemists and Biologists have extensively used colorimetric-based detection of toxins^1–3^, biological components^4–6^, explosives^7–9^, pollutants^10,11^, and nutrients^12,13^ both from commercial as well as environmental samples. These colorimetric sensors have been developed not only for their accurate identification through specific chemical interactions^7^ but also for their ability to detect in trace level in case of early stage detection of cellular components^6^ in a biological process as well as for a chemical compound^10,11^ as an active ingredient. Scientists throughout the globe have invested their time and invented several robust colorimetric sensors for heavy metals which include As(III and V), Pb(II), Hg(II), Cd(II), etc.^1,3,14^ by considering their severe health disorder activity. Compared to these toxic heavy metals, efficient colorimetric sensors for precious metals like Au, Ag, Pt, Pd, Ru, etc. are very rare in the literature and seek a proficient analytical assay for their accurate sensing and measurement. Most of these colorimetric detections are based on either the intensification or reduction of mother color on exposure of the analyte to the colorimetric sensor and the degree of color intensity change measures the extent of analyte present. Contrary to that, change in color based on decoupling of different plasmons offered by a nanoparticle upon exposure to a particular analyte (here gold-ion) at different concentrations to offer a color-coded sensing is probably the rarest report in the literature. Along with their insoluble pure metallic form, precious metals can also remain as salts which are highly soluble in water. Due to its attractive characteristics like, malleability, strong reflectivity in infrared region, good conductivity of heat and electricity, high density along with chemical resistivity, gold (Au) has widely been used as a precious metal in several fields such as jewellery, medicine, high-tech industries, catalysis, electrical & electronic industries and corrosion-resistant coatings^15–22^. Driven by the needs, here we have reported a highly selective (over other metal ions) and ultrasensitive (up to *ppt* level) decoupled *in-plane* dipole resonance modulated turn-on colorimetric assay-based optical ruler for accurate gold quantification. *In situ* generated silver nanodiscs (AgNDs) show different extent of red shifting of the *in-plane* dipole resonance (longitudinal mode) based on their increased particle size as we decrease the Au-ion concentration in the assay. In past, different instrumental techniques like, inductively coupled plasma mass spectrometry (ICP-MS), inductively coupled plasma optical emission spectrometry (ICP-OES), electrothermal atomic absorption spectrometry (ETAAS), energy-dispersive X-ray spectroscopy (EDX), flame atomic absorption spectrometry (FAAS), and spectrophotometry^23–31^ have been employed to detect the extent of Au present in a sample. Among them, ICP-MS and ICP-OES are the most sensitive techniques to detect very low level of Au content. However, due to their high cost, complicated instrumentation and time-consuming nature forced them not to be suitable for routine applications^24–26^.

Along with numerous sensitive detection techniques, several pre-concentration methods (e.g., ion exchange, membrane separation, co-precipitation, solvent extraction, cloud point extraction, and sorption on a solid phase) are also been available to make enriched Au samples for easy detection by adopting stable complexation mechanism^32–42^. Though they are simple and effective techniques, they suffer from high cost, time consuming nature, and use of large volume toxic solvents as extractants^43–45^. A rapid and selective Au-ion recognition and recovery was investigated recently by a new type of mesoporous adsorbent^46^, where the solution acidity plays an important role to bind trace level of Au-ion to these functional groups. The specificity and fast complexation of the metal ions with ligand immobilized conjugate adsorbent was investigated for selective and sensitive detection and recovery of Au-ions from waste scrap^47^. Different functional-group ligands trapped on a variety of solid matrices have also been used for sensitive determination of trace level Au-ions based on easy to use and cost effective adsorbents^48^.

The objective of the present study is to develop a low cost yet novel turn-on colorimetric sensor assay for highly specific and ultrasensitive detection of trace level gold content simply by controlling the extent of decoupling and thereby tuning the *in-plane* dipole resonance (longitudinal mode) of the *in situ* generated AgNDs. In presence of different concentration of Au-ion (*ppm* to *ppt* level), homogeneous mixture of sodium citrate tribasic dihydrate (TSC), silver nitrate (AgNO_3_) and L-ascorbic acid (AA) which we termed as ‘growth solution’ forms different sized AgNDs (along with spherical silver nanoparticles, AgNPs). Controlling the decoupling (of *in-plane* dipole resonance from *out-of-plane* dipole resonance) and thereby tuning the *in-plane* dipole resonance of these *in situ* produced AgNDs generate different complementary colors for efficient readout of the extent of gold present in the solution as impurity. In other words, by optical assay we can measure the extent of gold as impurity by observing the peak wavelength from the measured absorbance (or plasmon band) and the corresponding complementary color is detected by our colorimetric assay. As a result of this the turn-on colorimetric assay-based optical ruler can be used as a powerful tool for Au-ion detection as the appearance of color positively confirms the presence of gold as gold ion and the longitudinal plasmon (*in-plane* dipole resonance) tuning measures the extent of Au-ion present as tracer. A plausible mechanistic explanation, based on the relative oxidation/reduction potentials of active redox systems present in the sensor assay has been presented in great details that elucidates the efficient use of AA as a reducing agent catalyzed by Au-ion to reduce available soluble metal ions. Present study also investigates the extent of interference from foreign metal ions not only to measure the specificity of this sensor but also to understand the enhanced sensitivity and their stability with time. Finally, we have tested our assay to detect and measure gold concentration from real ore samples and to find out the possible interference of the matrix or whether we can use this interference to enhance the sensitivity of detection without disturbing the overall specificity of the assay.

## Methods

### Chemicals and Reagents

Chemicals, including silver nitrate (AgNO_3_; Bioxtra, 99%, (titration)), gold(III) chloride trihydrate(HAuCl_4_.3H_2_O; ≥99.9%, trace metals basis), sodium citrate tribasic dihydrate (C_6_H_5_Na_3_O_7_.2H_2_O; Bioultra, for molecular biology, ≥99.5% (NT)) and L-ascorbic acid (BioXtra, ≥99.0%, crystalline) were purchased from Sigma Aldrich and used without any further purification. All the glasswares used in the experiment was cleaned by the liquid detergent extran and then washed thoroughly with double distilled water and finally with Milli-Q water and dried in an oven before use. For all of the preparation steps we used Milli-Q water with a resistivity of 18.2 MΩcm.

### Growth Solution Preparation

Under continuous stirring condition 175 µL of 1% sodium citrate tribasic dihydrate (TSC), 250 μL of 10^−2^ M silver nitrate (AgNO_3_) and 50 µL of 10^−1^ M of ascorbic acid (AA) were added one-after-another into 10 mL of Milli-Q water. We named this homogeneous solution as ‘growth solution’.

### Synthesis of Silver Nanoparticles (AgNPs)

**Figure 1:**
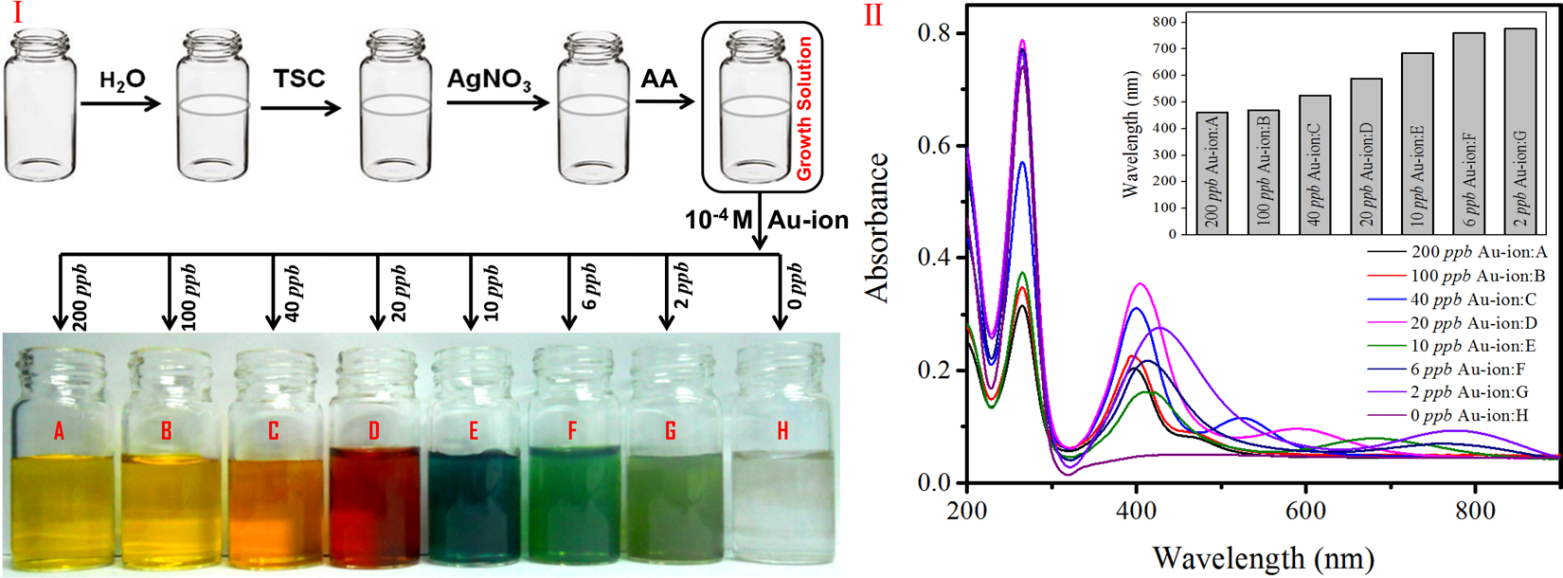
(**I**) A stepwise Au-ion catalyzed AgNDs synthesis process at room temperature in TSC medium by reducing AgNO_3_ in presence of ascorbic acid and (**II**) UV-vis spectra of *in situ* generated AgNPs (mixture of nanospheres and nanodiscs) synthesized at different concentrations of Au-ion. The spectra show one strong peak at 265.5 nm and a small hump (Fig. 1S) near 335 nm due to the absorption of AA and *out-of-plane* quadrupole resonance of AgNDs respectively. The *out-of-plane* dipole resonance of AgNDs and both the *in-plane* and *out-of-plane* dipole resonances of AgNPs (nanospheres) give rise to a peak near 400 nm. Another tunable band between 500 and 800 nm originates from the *in-plane* dipole resonance of AgNDs. The appearance of *in-plane* dipole resonance from individual AgNDs synthesized at different gold concentration is shown as bar plot in the inset.

Under continuous stirring condition different concentration of Au-ion was added to the ‘growth solution’ and the whole solution turned into colored solution within few minutes, indicating the formation of silver nanoparticles (AgNPs). The reaction was carried out at room temperature in a 15 mL Borosil glass bottle placed on a magnetic stirrer allowing continuous mixing of chemicals. The reaction was allowed to proceed for another ~2 min after the addition of all reagents simultaneously. We also added different metal ion to the solution to see their influence in AgNP formation. The schematic representation of the stepwise synthetic procedure of the Au-ion catalyzed AgNP synthesis has been explained in Fig. 1(I) and their corresponding plasmon peak appearance due to the production of Au-ion catalyzed shape and size variable AgNPs has been presented in Fig. 1(II) with detail description in the later section. Associated controlled experiments to confirm the necessity of gold ion’s presence has been tested in a systematic way. Influence of other heavy metals and alkali metals for the possible false positive sensing or for the improvement of the sensitivity of gold detection has also been discussed in details in the result and discussion section.

### Spectroscopic Characterization

The absorption spectra of dispersed AgNPs were measured with an UV–vis spectrophotometer Jasco V650 at room temperature (25 °C).

### Electron Microscopic Characterization

Confirmations of AgNDs formation, their size variation, and crystallographic information of the generated silver nanoparticles in presence of different concentrations of Au-ions have been done by TEM characterization. For High Resolution Transmission Electron Microscopic (HRTEM) measurements we used a FEI, Tecnai G^2^F30, S-Twin microscope operating at 300 kV. Details of TEM measurement have been included in the supporting information section.

#### Extraction of Gold as Gold Ion from the Ore

Soluble gold extraction has been done by treating 0.01 g of oxidized ore with 100 µL of 10-times diluted aqua regia for 10 min and then the extracted solution was centrifuged at 12000 rpm for 10 min to remove any insoluble materials. Top decanted solution after centrifugation was diluted 10 times further and used as stock solution for the entire sensing experiments.

## Results and Discussion

Reagents which are frequently used to reduce Ag^+^ to Ag^0^ include N,N-dimethylformamide, hydrazine, ferric ions (Fe^3+^), and sodium borohydride. Unfortunately, most of these reducing agents exhibit undesired toxicity, which limit their application in medical and packaging products. In this study, ascorbic acid (chemical name of vitamine C which is non toxic) has been employed for the reduction of silver ions to form a mixture of spherical- and disc-shaped (AgND) silver nanoparticles. In the present set of demonstration, presence of trace level Au-ion is necessary to generate these AgNPs. This observation is clearly demonstrated in Fig. 1(I), where the right most bottle with 0 *ppb* concentration of Au-ion did not produce any color and hence confirms the absence of any plasmonic nanoparticle formation. In presence of different concentration of Au-ions (200 *ppb* to 2 *ppb*), the ‘growth solution’ tuned its color from yellow to light green which has been explained by considering the formation of different sized AgNDs.

**Figure 2:**
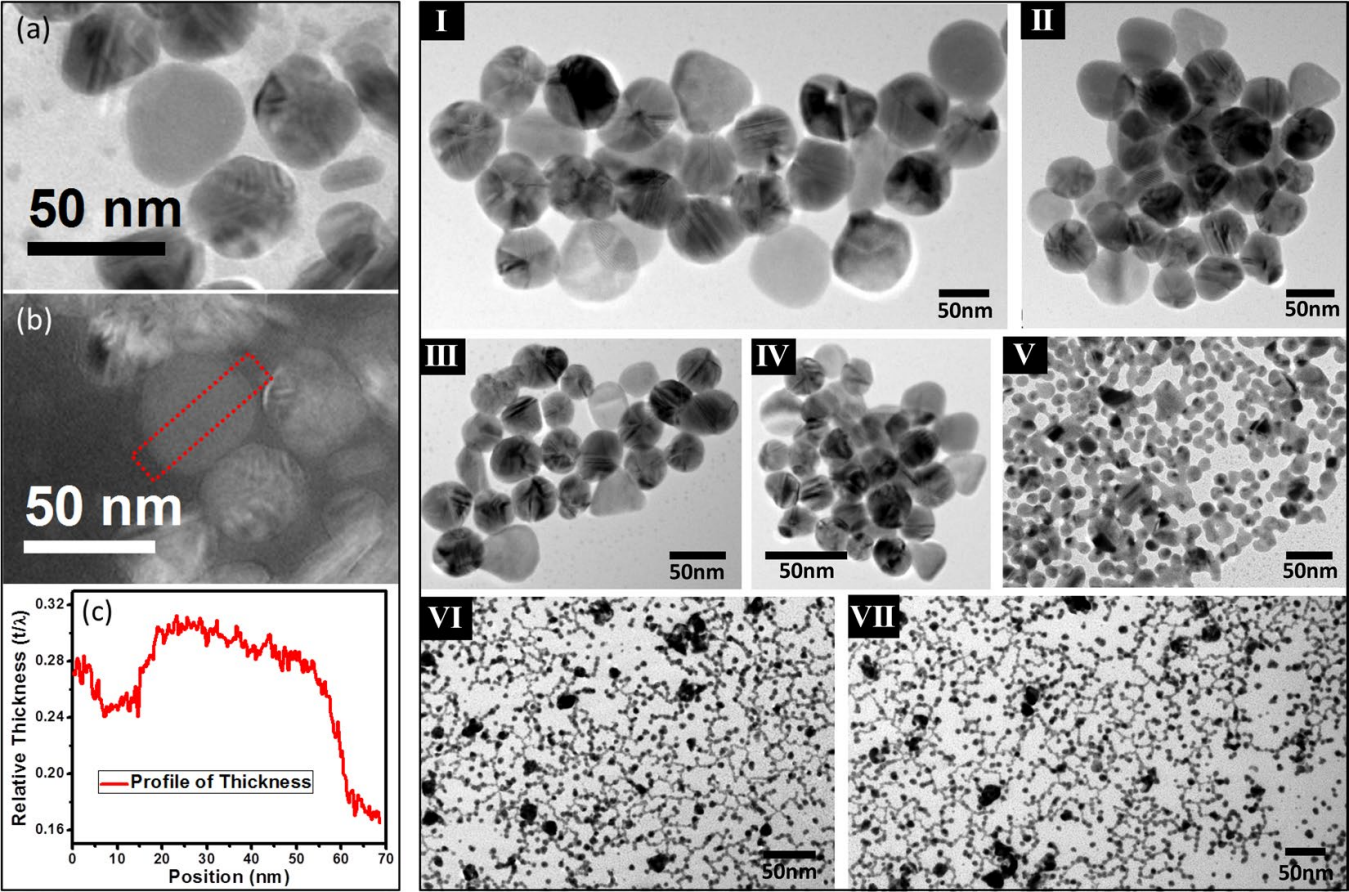
(**a–c**) Energy filtered TEM images: (**a**) Elastic image, (**b**) Relative thickness map, (**c**) Average thickness profile over dotted area marked in (**b**) showing flat-top morphology. From the figure (**c**) the relative thickness of nanodisc is about 0.065 which corresponds to about 9.75 nm (by considering λ at 150 nm), the average thickness of the nanodisc. (I)–(VII) present TEM images of size variable AgNDs with spherical AgNPs present in mixture using different concentration of catalytic gold (I: 2 *ppb*, II: 6 *ppb*, III; 10 *ppb*, IV: 40 *ppb*, V: 200 *ppb*, VI: 50 *ppm*, VII: 200 *ppm*).

The UV–vis absorption spectra of the turn-on colorimetric sensor assay in presence of different concentration of Au-ion (sample A-G) and in absence of Au-ion (Sample H) at room temperature in aqueous medium was recorded and presented in Fig. 1(II). The absorption spectrum of the sample H doesn’t show any surface plasmon band rather absorbs strongly at 265.5 nm due to the presence of AA. For samples A-G, in addition to this 265.5 nm peak they show a small hump near 335 nm (Fig. 1S) and additional two surface Plasmon absorption bands, one near 400 nm with smaller tunability between 397 nm and 427 nm and another tunable band between 500 nm and 800 nm. Surface plasmon resonances are unevenly distributed around non-spherical metallic nanoparticles and this demonstrates their shape dependent localized surface plasmon resonance spectra^49,50^. For spherical silver nanoparticles both the *in-plane* and *out-of-plane* dipole plasmon resonances appear in the same energy and hence remain coupled in the extinction spectra to demonstrate a single intense sharp peak near 400 nm. When silver nanoparticles change their shape from spherical to two dimensional structures, *in-plane* and *out-of-plane* resonances start appearing in different energy range of the electromagnetic spectrum and decouples from each other. Despite forming spherical particles, decoupling of plasmons in the present study occurs mainly due to the formation of two dimensional AgNDs and depending on their dimension the *in-plane* dipole resonance tunes over a wide range (between 500 nm and 800 nm). According to the previous reports^51–54^ the appearance of the hump near 335 nm is due to the *out-of-plane* quadrupole resonance, surface plasmon band near 400 nm originates from *out-of-plane* dipole resonance (transverse mode), and *in-plane* dipole resonance (longitudinal mode) band varies between 500 nm and 800 nm depending on the diameter and thickness of the *in situ* generated AgNDs. Expected *in-plane* quadrupole resonance band near 470 nm^51–53^ does not appear as a clear band and could be enveloped inside the *out-of-plane* dipole resonance to make it difficult to decouple. Two times increment (from 60 nm to 130 nm) of full width at half maxima (FWHM) by moving the particle diameter from 5 nm to 50 nm indirectly proves our assumption. It is also noticeable from the absorption spectra that irrespective of the size and shape of the generated AgNPs, dipole resonances (*in-plane* and *out-of-plane*) are always more intense compared to their corresponding quadrupole resonances which is expected from their transition probabilities^50^. When the particle size is very small (below 5 nm), *in-plane* and *out-of-plane* dipole resonances appear close to each other and intend to merge into a single peak. Contrary to that as the particle size increases, the *in-plane* and *out-of-plane* dipole resonances start decoupling from each other depending on their diameter and thickness. As the diameter of the particle increases, both the *out-of-plane* and the *in-plane* dipole resonances red shifted with respect to their smaller particle counterparts. In case of *out-of-plane* dipole resonance the extent of red shifting is only 30 nm (between 397 nm and 427 nm) whereas for *in-plane* dipole resonance the extent of red shifting shows a much wider plasmon tuning of 313 nm (between 463 nm and 776 nm). The kinetics of the structural transition and the stability of the so-produced AgNDs was monitored by real-time UV-vis spectroscopy (Fig. 2S).

It is clear from the recent study of O’Brien *et al*.^53^ that a very thin disc (below 10 nm thickness) does not generate any noticeable *out-of-plane* resonance band due to the lack of oscillating free electron in the perpendicular direction to support a plasmon. So the intensity of *out-of-plane* dipole resonance indirectly measures the thickness whereas the intensity of *in-plane* dipole resonance measures the diameter of the disc and aspect ratio of the disc controls the decoupling and hence tuning of their plasmons. Disc natures of the *in-situ* generated nanoparticles are clearly shown in Fig. 2(a–c) by recording their energy filtered TEM images^55^. It is clear from our energy filtered TEM image for Sample-F (of Fig. 1(I) with 6 *ppb* Au-ion) that the measured average thickness profile over the entire nanoparticle is showing flat-top morphology with an average thickness of ~9.75 nm which should not be able to support an *out-of-plane* dipole resonance. Hence the entire intensity of *out-of-plane* dipole resonance comes from *in situ* generated spherical AgNPs.

**Figure 3:**
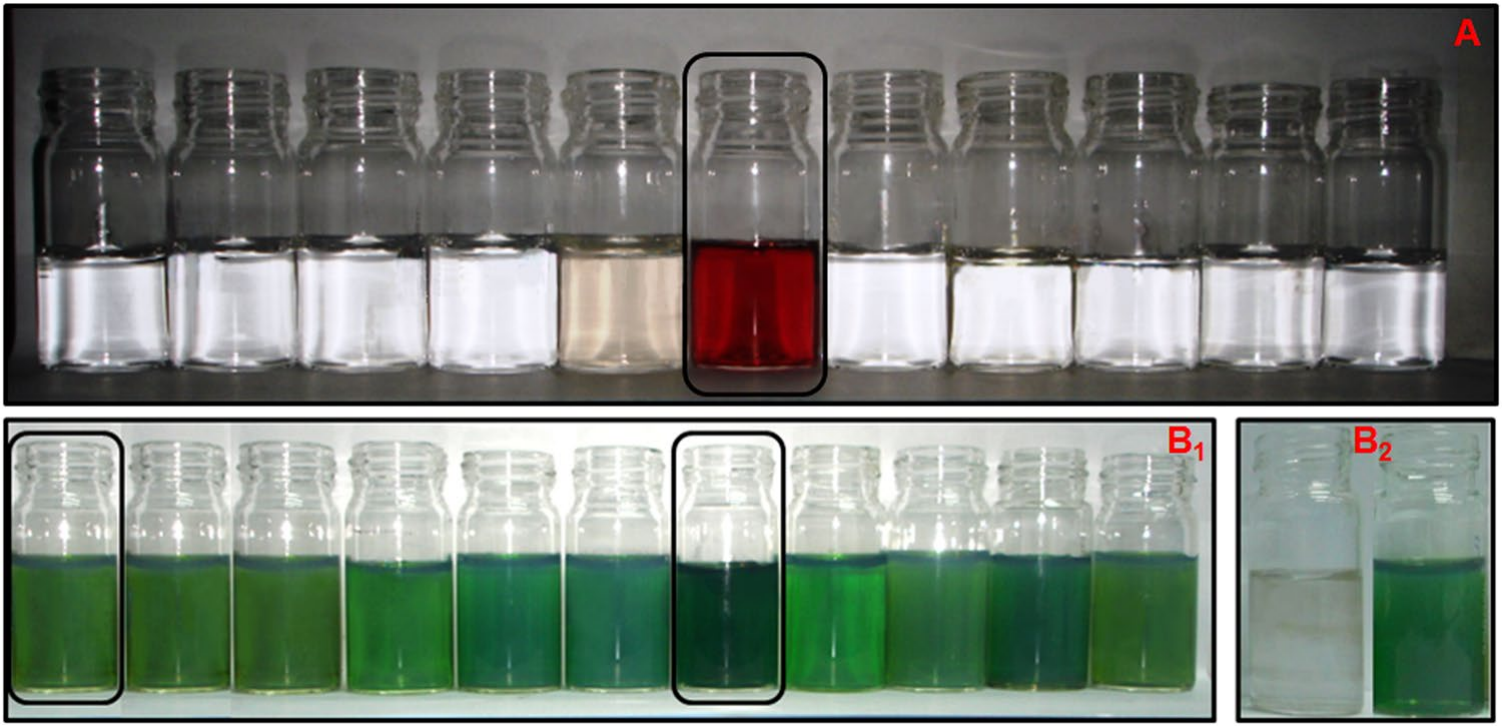
(**A**) Selective Au-ion sensing by the ‘growth solution’ in presence of different metal ions (600 *ppb*). From left to right: Na^+^, Pd^4+^, Ru^3+^, As^3+^, Hg^2+^, Au^3+^ (20 *ppb*), Mg^2+^, Zn^2+^, Cd^2+^, Pb^2+^, and Cr^3+^. (**B**_**1**_) Effect of different interfering metals (600 *ppb*) in presence of 2 *ppb* Au(III). From left to right: Blank (2 *ppb* Au(III)), Na^+^, Pd^4+^, Ru^3+^, As^3+^, Mg^2+^, Hg^2+^, Zn^2+^, Cd^2+^, Pb^2+^, and Cr^3+^. (**B**_**2**_) Effect of Hg^2+^ to enhance the detection limit of Au tracer. From left to right: ‘Growth solution’ in presence of 700 *ppt* Au(III) without 600 *ppb* Hg^2+^ and with 600 *ppb* Hg^2+^.

The extent of plasmon shifting for a nanodisc to that of a nanosphere^56^ by applying same optical force has been calculated analytically in the Supporting Information Section where variation of relative stress for discs (with a constant disc thickness) and spheres with their diameter have been estimated (Fig. 3S). The result shows that the induced stress directly depends on their aspect ratios.

By any way we don’t mean to establish this synthetic protocol as a methodology to generate AgNDs exclusively rather provides an easy colorimetric assay-based optical ruler technique for the trace-level quantitative gold detection simply by decoupling *in-plane* dipole resonance from their *out-of-plane* dipole resonance. As it is clear from the obtained TEM images that the gold-catalyzed silver nanomaterials synthesis generates both solid spherical particles as well as disc particles, it is expected to observe both *in-plane* and *out-of-plane* dipole resonances in their recorded UV-vis spectra where the *in-plane* dipole resonance contribution comes mainly from silver nanodiscs and *out-of-plane* contribution mostly comes from spherical silver nanoparticles. Relative intensity of *out-of-plane* and *in-plane* resonance measures their abundance and it is also clear from the UV-vis spectra that the extent of shperical particles formation is always much more compared to the nanodiscs.

The UV-vis peak at 265.5 nm indicates the presence of unreacted AA present in the solution and its intensity acts as a ruler to measure the catalytic efficiency of Au-ion to force AA to act as an efficient reducing agent. When we vary the final concentration of Au-ion in our assay between 200-2 *ppb*, an increment of the size of the generated AgNPs (between 5–50 nm) has been observed as evident from their UV-vis spectra (Fig. 1(II)) as well as from TEM images (Fig. 2(I–VII)). Details of TEM measurement is described in the Supporting Information Section. From Fig. 1(II) it is also clear that the plasmon peak responsible for *out-of-plane* dipole resonance which indirectly measures the diameter of the generated spherical nanoparticles moves from 427 nm to 397 nm as we vary the added gold ion concentration between 2–200 *ppb*. Compared to the *out-of-plane* dipole resonance, the *in-plane* dipole resonance which measures the dimension of the AgNDs tuned between 800 nm and 500 nm by varying the Au-ion concentration in the same range.

**Figure 4:**
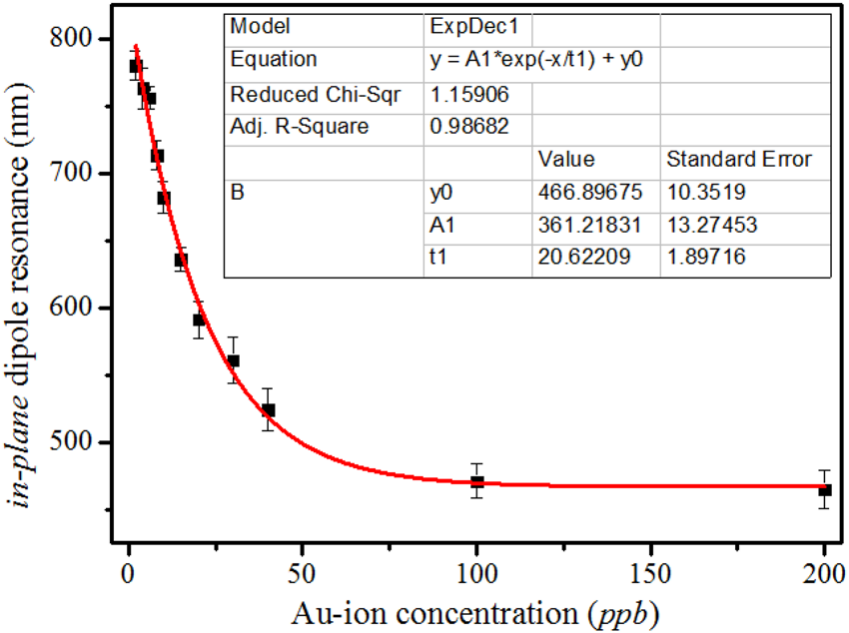
Variation of *in-plane* dipole resonance with the concentration of added Au-ion where the plotted *in-plane* dipole resonances are in nm and the gold ion concentrations are in part-per-billion.

**Figure 5:**
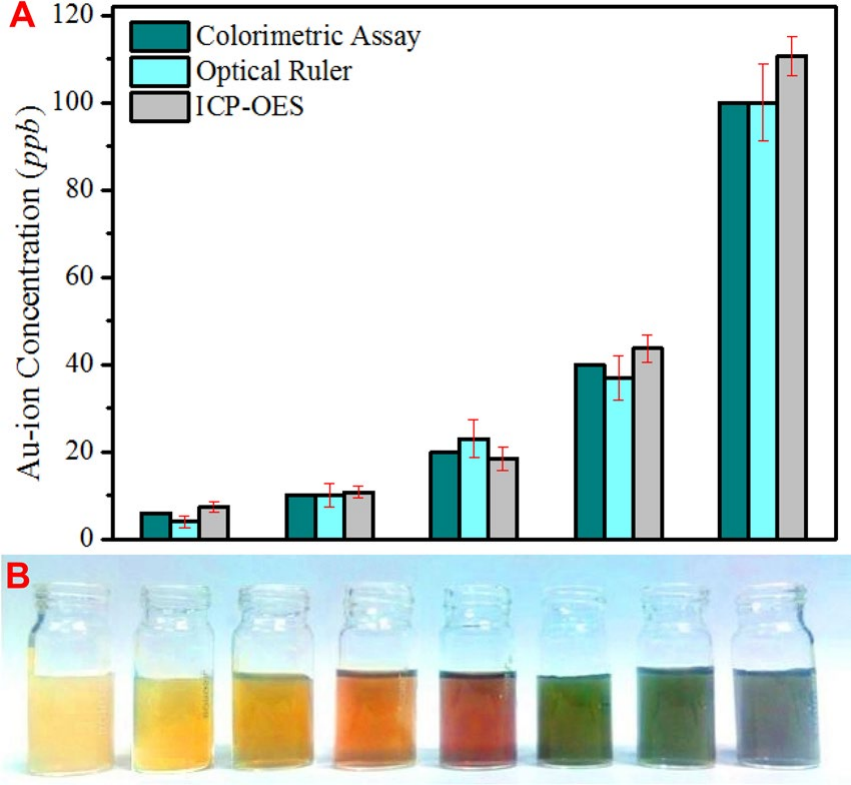
(**A**) A Comparative sensitivity analysis of Au-ion detection by our colorimetric and optical assay with respect to the ICP-OES as standard analytical sensing tool is shown as bar plots. Average error both for colorimetric assay and optical ruler is estimated about ~12%. (**B**) Colorimetric response from oxidized ore samples as we vary the concentration of gold ion (L-R: 300, 210, 150, 120, 90, 60, 30 and 18 *ppt*).

To avoid any ambiguity of color coding, we have conducted several controlled experiments which include: (i) if we add variable amount of Au-ion solution in absence of TSC which acts as a surfactant in the composition (acts as a reducing agent only at elevated temperature) of ‘growth solution’, we have not observed any color appearance or color change, (ii) in absence of AA which acts a reducing agent (acts also as a stabilizing agent^57^), we could not achieve the reduction of Ag^+^ and the subsequent formation of AgNDs to result color coding, (iii) in absence of both TSC and AA, formation of AgNDs is prohibited, and (iv) even in absence of TSC and AgNO_3_ (to prove that the appearance of color is not due to the reduction of Au-ions by AA) there is no appearance of color. *In situ* generation of AgNDs are achieved only when all the essential component of the ‘growth solution’ with the optimal concentration (i.e. 5.3 × 10^−4^ M TSC, 2.5 × 10^−4^ M of AgNO_3_, and 5 × 10^−4^ M of AA) reacts with an aqueous solution containing variable amount (200–2 *ppb*) of Au-ions and results different distinct colors. To confirm the fact further that the color of the growth solution is originating from AgNPs only, we performed another set of controlled experiments by varying the gold-ion concentration from 2 *ppb* to 1000 *ppb* in absence of silver nitrate solution while keeping other ingradients (TSC, AA) present in the above mentioned concentrations. Details of these control experiments have been described in Fig. 4S. It is known in the literature^57^ that AA can effectively act both as a stabilizing agent as well as reducing agent to generate gold nanoparticles. To cross check this, we have performed the same set of control experiments at higher concentration of Au-ion (>200 *ppb*) which generate nanoparticles predominated by gold nanoparticles and the subsequent color varies from yellow to red and finally to brown with a strong plasmon near 500 nm, a characteristic plasmon peak for gold nanoparticles. Effect of higher concentration of Au-ion to the ‘growth solution’ is described in Fig. 5S. Figure 5S also explains that the presence of Hg^2+^ (HgCl_2_) which enhances the detection sensitivity of Au by three times (×3), alone can’t produce any color change; rather generates a gray color originating from insoluble AgCl. Reported EDX data (Figure 6S) show trace of Au only when the added Au-ion concentration is above 40 *ppb*.

Controlled experiments show the unusual and striking role of trace level Au-ions for the formation of AgNDs and offer a reliable methodology for highly specific sensing of different level of Au tracer. The role of trace level Au-ion can be explained by considering the reduction potentials of the component redox systems (a. Au^3+^/Au^0^, b. Ag^+^/Ag^0^ and c. AA^2+^/AA) present in the ‘growth solution’. Reduction potentials for Au^3+^/Au^0^, Ag^+^/Ag^0^ and AA^2+^/AA are 1.5, 0.81 and −0.066 V (at pH 7) respectively^58–61^. In absence of Au-ions, when the system is loaded by Ag^+^ and AA, comparison of reduction potentials indicates that the available Ag^+^ ions should be reduced by AA due to more + ve reduction potential of Ag^+^/Ag^0^ system and those electrons should be supplied by AA. Oxidation potential of AA is only +0.066 V and hence the differential reduction potential (between Ag^+^/Ag^0^ and AA^2+^/AA) of ~0.8 V results a very low tendency to lose electrons to reduce Ag^+^ for AgND formation and a large portion of AA (signifies the absorbance at 265.5 nm) remains un-reacted as evident from Figure 7S. Presence of Au-ion changes the situation dramatically as the reduction potential of Au^3+^/Au^0^ is substantially higher than Ag^+^/Ag^0^ and the resultant differential reduction potential (between Au^3+^/Au^0^ and AA^2+^/AA) of 1.5 V forces AA to acts as an efficient reducing agent and results all the available AA being used in the reduction process (disappearance of 265.5 nm peak from UV-vis spectra). Efficient use of AA as a reducing agent catalyzed by Au-ion to reduce available metal ions present in the ‘growth solution’ is clearly observable from Figure 7S.

So, here Au-ions act as a catalyst to initiate the ascorbic acid oxidation process and the released electrons are mostly captured by silver ions due to 1000-fold more concentration of silver ions present in the solution compared to Au-ions. Observed EDX data (Figure 6S) show that the generated nanoparticles are composed by silver only which alternatively proves our argument.

Main challenge for any potential sensor is its selectivity towards the targeted one over competitive impurities present in the system. To show the specificity of this ‘growth solution’ towards Au-ions, we have tested this novel sensor in a multi elemental environmental sample. Preparation of the ‘growth solution’ remains same as we explained before and then added 600 *ppb* of different metal salts (Na^+^, Pd^4+^, Ru^3+^, As^3+^, Hg^2+^, Mg^2+^, Zn^2+^, Cd^2+^, Pb^2+^, and Cr^3+^) individually instead of Au salt to this ‘growth solution’ under constant stirring. We have not tested Pt as most of their salts are insoluble in water. As it is clear from Fig. 3A that, in absence of Au-ions none of these samples (contaminated by different salts) are capable to generate AgNDs and thereby produce any visible change (Hg^2+^ gives gray color originating from insoluble AgCl) in the solution color for easy colorimetric detection. This proves the necessity of trace level of Au-ion for efficient formation of AgNDs.

To find out the interfering aptitude of the other metals while gold present in the system, we have tested the sensing ability of our ‘growth solution’ by adding 2 *ppb* of Au(III) with 600 *ppb* of other metal salt solution individually or collectively. Except mercury, none of the other metal salts show much noticeable effect observable by simple colorimetric change. Worthwhile to mention that the presence of mercury (along with Au) doesn’t change the color, plasmon resonance or the characteristics of the synthesized AgNDs rather it intensifies the color compared to the assay with equal amount of Au-ion without mercury impurity as clearly observable from Fig. 3B_1_. Presence of 600 *ppb* of Hg^2+^ enhances the Au-ion detection limit by three times and we could easily detect up to 700 *ppt* concentration. Effect of 700 *ppt* Au-ion in presence of 600 *ppb* of Hg^2+^ ions is shown in Fig. 3B_2_. Maximum interference limit for other metal ions achieved from our experiment is 1 *ppm*.

Quantification of Au-ion can easily be achieved either from the observed color change or from the measured in-plane dipole resonance. Variation of *in-plane* dipole resonance with the concentration of added Au-ion has been plotted in Fig. 4 which shows an exponential fitting. We have repeated this detection several times (due to its low cost and one step detection nature) and gives absolutely reproducible result both spectroscopically as well as colorimetrically.

To find the universal applicability of our turn-on colorimetric sensor, we have verified the detection and quantification ability of our assay by performing colorimetric test with cross examination by standard ICP-OES detection of available Au present in the assay. As an unknown gold sample we have taken trisodium citrate (TSC)-based gold nanoparticle solution with unknown concentration but doesn’t have contamination of other metals except gold. 10 μL of this gold nanoparticle solution first evaporated to dryness and then dissolved in 10 μL of a 5-time diluted aqua regia solution as Au-ions, which further diluted to 1 mL to get the stock solution. From this stock solution we have taken 5, 10, 25, 50 and 90 μL of dissolved gold solution and mixed with our ‘growth solution’, which generates light green, deep green, deep red, orange and yellow color with tunable *in-plane* dipole resonance appearing at 759, 687, 583, 526 and 465 nm respectively. These specific colors correspond to the Au-ion concentration of 6, 10, 20, 40 and 100 *ppb* from our known colorimetric assay (Fig. 1(I)) and 4, 10, 23, 37 and ~100 *ppb* from our optical assay as depicted in Fig. 4. We studied these aqueous phases obtained from our turn-on colorimetric assay by ICP-OES to measure the actual Au-ion concentration. For ICP-OES calibration, a 1000 *ppm* Au solution from NIST was used as known standard. Details of the employed operating conditions are listed in Table 1S. Obtained gold concentration from the ICP-OES measurement for the above five solutions are 7.365, 10.8, 18.392, 43.7 and 110.61 *ppb* respectively that matches well with our expected values (average error: ~12% which is obtained by calculating the % of error in Au-ion concentration measurement both from colorimetric and optical assay with respect to the standard ICP-OES method and then taking a final average of all detections. Details about the % of error calculation has been included in the supplimentary information section.) and proves the robustness of our turn-on colorimetric assay for accurate Au-ion concentration measurement suitable for field applications both in mining industry as well as in forensic science. A comparative sensitivity of Au-ion detection by our colorimetric assay-based optical ruler with respect to the ICP-OES as standard analytical sensing tool is shown in Fig. 5A.

For any reliable sensor, the major concern is the possible interference of the matrix for real sample detection. To prove the reliability of our technique, we have verified our assay further by testing real environmental samples. We have received (from MISiS, Moscow, Russia) an oxidized gold ore and its tailing (Bugunda Gold Deposit, Tajikistan) with average gold content of 6 g/ton and 3 g/ton respectively. We have extracted gold from the oxidized ore by converting metallic gold (Au^0^) into soluble gold (Au^3+^) by dissolving the ore into aqua regia solution. To avoid the effect of pH on the detection efficiency we have pre-diluted the aqua regia which is sufficient to dissolve the deposited gold in the ore but the pH of the “growth solution” remain neutral after the addition of gold impurity. Due to the presence of several other heavy metals as co-minerals (silver, copper, iron, mercury, arsenic, etc.) in the ore, solubilization of gold ore also solubilize other metals as ions and hence the resultant ore solution is a mixture of several different metal ions. As mentioned before that the detection efficiency highly depends on the concentration of mercury ion as impurity in the gold solution, contamination by several other metal tracer may influence the detection efficiency several orders compared to the ideal case as we have explained for pure gold ion detection. Details about the soluble gold extraction from oxidized ore and tailing has been described in the experimental section. We have taken 3, 5, 10, 15, 20, 25, 35 and 50 µL of the extracted stock solution from oxidised ore and added to the “growth solution” which generates gray, light green, deep green, red, orange and different shades of yellow color respectively after few minutes of treatment as has been shown in Fig. 5B. By considering the initial gold content of 6 g/ton (Confirmed by ICP-OES) for oxidized ore, the final concentration corresponding to different colors are 18 *ppt*: gray, 30 *ppt*: light green, 60 *ppt*: deep green, 90 *ppt*: red, 120 *ppt*: orange, 150 *ppt* and above: different shades of yellow. The detection limit (30 *ppt* concentration shows the first colorimetric signature) obtained from the oxidized gold ore sample is thus at least 10 times more sensitive compared to the pure gold or gold with mercury samples. Despite the more sensitivity of gold detection from ore sample, the pattern of color change with increasing concentration of gold content remains unchanged and this more sensitivity of gold detection could be due to the presence of innumerable different metals and nonmetals present in the ore samples which in general gives more sensitivity with unchanged specificity as has been described for Hg(II) impurity of gold sample.

### Associated Content

Details about growth solution preparation, nanoparticle characterization, assignment of *out-of-plane* quadrupole resonance, kinetics of the growth of the AgNDs, analytical calculation of relative stress for a disc and a sphere, controlled experiments including operating parameters of the ICP-OES used for the measurement of Au-ion tracer, EDX spectra and role of Au-ion as catalyst are included in the Electronic Supporting Information section.

## Conclusion

In summary, present study reports the development of a turn-on colorimetric sensor assay for highly specific and ultrasensitive detection and quantification of gold (Au) content in a multi-elemental environmental sample (gold ore) with achievable sensitivity way down to 30 *ppt*. Detection and quantification of gold was achieved by a low cost green synthetic route mediated decoupled *in-plane* dipole resonance modulated colorimetric assay-based optical ruler of *in situ* generated variable sized AgNDs. We have put forward a plausible explanation of the role of Au-ion tracer to initiate the shape variable silver nanoparticle formation in the ‘growth solution’ by considering the reduction potentials of the component redox systems. By considering the cost, toxicity, and simplicity to perform a sensing experiment, our turn-on colorimetric sensor offers a robust analytical assay for accurate gold (Au) concentration measurement suitable for field applications both in mining industry as well as in forensic science.

## Electronic supplementary material

Supplementary Information

## References

[1] Kalluri JR (2009). Use of gold nanoparticles in a simple colorimetric and ultrasensitive dynamic light scattering assay: selective detection of arsenic in ground water. Angew. Chem. Int. Ed..

[2] Lim SH, Feng L, Kemling JW, Musto CJ, Suslick KS (2009). An optoelectronic nose for the detection of toxic gases. Nat. Chem..

[3] Beqa L (2011). Gold nano-particle based simple colorimetric and ultrasensitive dynamic light scattering assay for the selective detection of Pb(II) from paints, plastics, and water samples. ACS Appl. Mater. Interfaces.

[4] Lu W (2010). Multifunctional oval-shaped gold-nanoparticle-based selective detection of breast cancer cells using simple colorimetric and highly sensitive two-photon scattering assay. ACS Nano.

[5] Valentini P (2013). Gold-nanoparticle-based colorimetric discrimination of cancer-related point mutations with picomolar sensitivity. ACS Nano.

[6] Wang S (2010). Rapid colorimetric identification and targeted photothermal lysis of *Salmonella* bacteria by using bioconjugated oval-shaped gold nanoparticles. Chem. Eu. J..

[7] Dasary SSR, Singh AK, Senapati D, Yu H, Ray PC (2009). Gold nanoparticle based label-free SERS probe for ultrasensitive and selective detection of Trinitrotoluene. J. Am. Chem. Soc..

[8] Uzer A, Can Z, Akin I, Ercag E, Apak R (2014). 4-Aminothiophenol functionalized gold nanoparticle-based colorimetric sensor for the determination of nitramine energetic materials. Anal. Chem..

[9] Idros N (2015). Colorimetri-based detection of TNT explosives using functionalized silica nanoparticles. Sensors.

[10] Kim YR, Mahajan RK, Kim JS, Kim H (2010). Highly sensitive gold nanoparticle-based colorimetric sensing of mercury(II) through simple ligand exchange reaction in aqueous media. ACS Appl. Mater. Interfaces.

[11] Bai W (2015). Gold nanoparticle-based colorimetric aptasensor for rapid detection of six organophosphorous pesticides. Environ. Toxicol. Chem..

[12] He P, Shen L, Liu R, Li Z (2011). Direct detection of β-agonists by use of gold nanoparticle-based colorimetric assays. Anal. Chem..

[13] Chen YC, Lee IL, Sung YM, Wu SP (2013). Triazole functionalized gold nanoparticles for colorimetric Cr^3+^sensing. Sensors and Actuators.

[14] Darbha GK (2008). Selective detection of mercury (II) ion using nonlinear optical properties of gold nanoparticles. J. Am. Chem. Soc..

[15] Shaw CF (1999). Gold-based therapeutic agents. Chem. Rev..

[16] Rao CRM, Reddi GS (2000). Platinum group metals (PGM),occurrence, use and recent trends in their determination. Trends Anal. Chem..

[17] Cui J, Zhang L (2008). Metallurgical recovery of metals from electronic waste: areview. J. Hazard. Mater..

[18] Nakbanpote W, Thiravetyan P, Kalambaheti C (2002). Comparision of gold adsorption by Chlorella Vulgaris, ricehusk and activated carbon. Miner.Eng..

[19] Laatikainen M, Paatero E (2005). Gold recovery from chloride solutions with XAD-7: competitive adsorption of Fe(III) and Te(IV). Hydrometallurgy.

[20] Song J, Wang L, Zibart A, Koch C (2012). Corrosion protection of electrically conductive surfaces. Metals.

[21] Changmei S (2011). Resin with high adsorption selectivity for Au(III): preparation, characterization and adsorption properties. Chem. Eng. J..

[22] Murphy CJ (2008). Gold nanoparticle in biology: beyond toxicity to cellular imaging. Acc. Chem. Res..

[23] Kumeria T, Santos A, Losic D (2013). Ultrasensitive nanoporous interferometric sensor for label-free detection of gold(III) ions. ACS App. Mater. Inter..

[24] Calle IDL (2011). Ultrasound-assisted extraction of gold and silver from environmental samples using different extractants followed by electrothermal-atomic absorption spectrometry. Microchem. J..

[25] Liang P, Zhao E, Ding Q, Du D (2008). Multiwalled carbon nanotubes microcolumn preconcentration and determination of gold in geological and water samples by flame atomic absorption spectrometry. Spectrochim. Acta.

[26] Hassan J, Shamsipur M, Karbasi MH (2011). Single granular activated carbon microextraction and graphite furnace atomic absorption spectrometry determination for trace amount of gold in aqueous and geological samples. Microchem. J..

[27] Amin AS (2010). Utility of solid phase extraction for spectrophotometric determination of gold in water, jewel and ore samples. Spectrochim. Acta A Mol. Biomol. Spectrosc..

[28] Jarvis I, Totland MM, Jarvis KE (1997). Assessment of Dowex1-X8-based anion-exchange procedures for the separation and determination of ruthenium, rhodium, palladium, iridium, platinum and gold in geological samples by inductively coupled plasma mass spectrometry. Analyst.

[29] Elci L, Sahan D, Basaran A, Soylak M (2007). Solid phase extraction of gold(III) on Amberlite XAD-2000 prior to its flame atomic absorption spectrometric determination. Environ. Monit. Assess..

[30] Iglesias M, Anticó E, Salvadó V (1999). Recovery of palladium(II) and gold(III) from diluted liquors using the resin Duolite GT-73. Anal. Chim. Acta.

[31] Moawed EA, El-Shahat MF (2013). Synthesis, characterization of low density polyhydroxy polyurethane foam and its application for separation and determination of gold in water and ore samples. Anal. Chim. Acta.

[32] Chen S, Zhu X (2010). Simplified cloud point extraction-inductively coupled plasma mass spectrometry for the preconcentration/analysis of ultra-trace gold. Miner. Eng..

[33] Sen S, Seyrankaya A, Cilingir Y (2005). Coal-oil assisted flotation for gold recovery. Miner. Eng..

[34] Kinoshita T (2006). Sep. Purif. Technol..

[35] Soleimani M, Kaghazchi T (2008). The investigation of the potential of activated hard shell of apricot stones as gold absorbents. J. Ind. Eng. Chem..

[36] Tuzen M, Saygi KO, Soylak MJ (2008). Novel solid phase extraction procedure for gold(III) on Dowex M 4195 prior to its flame atomic absorption spectrometric determination. Hazard. Mater..

[37] Tavakoli L (2008). Development of cloud point extraction for simultaneous extraction and determination of gold and palladiumusing ICP-OES. J. Hazard. Mater..

[38] Qu R (2009). Adsorption of Au(III) from aqueous solution using cotton fiber/chitosan composite adsorbents. Hydrometallurgy.

[39] Ertan E, Gulfen M (2009). Separation of gold(III) ions from copper(II) and zinc(II) ions using thiourea-formaldehyde or urea-formaldehyde chelating resins. J. Appl. Polym. Sci..

[40] Koyanaka H, Takeuchi K, Loong CK (2005). Gold recovery from parts-per-trillion-level aqueous solutions by a nanostructured Mn_2_O_3_adsorbent. Sep. Purif.Technol..

[41] Gomes CP, Almeida MF, Loureiro JM (2001). Gold recovery with ion exchange used resins. Sep. Purif. Technol..

[42] Sanchez JM, Hidalgo M, Salvado V (2001). Synthesized phosphine sulphide-type macroporous polymers for the preconcentration and separation of gold (III) and palladium (II) in a column system. React. Funct.Polym..

[43] Akita S, Yang L, Takeuchi H (1996). Solvent extraction of gold(III) fromhydrochloric acid medium by nonionic surfactants. Hydrometallurgy.

[44] Kilic AG, Malci S, Celikbicak O, Sahiner N, Salih B (2005). Gold recovery onto poly(acrylamide-allythiourea) hydrogelssynthesized by treating with gamma radiation. Anal. Chim. Acta.

[45] Fujiwara K, Ramesh A, Maki T, Hasegawa H, Ueda K (2007). Adsorption of platinum (IV), palladium (II) and gold (III) from aqueous solutions onto L-lysine modified crosslinked chitosan resin. J. Hazard. Mater..

[46] Awual MR, Khaleque MA, Ferdows M, Chowdhury AMS, Yaita T (2013). Rapid recognition and recovery of gold (III) with functional ligand immobilized novel mesoporous adsorbent. Microchem. J..

[47] Awual MR, Ismael M (2014). Efficient gold(III)detection, separation and recovery from urban mining waste using a facial conjugated adsorbent. Sensors and Actuators.

[48] Awual MR (2014). Investigation of potential conjugate adsorbent for efficient ultra-trace gold(III) detection and recovery. J. Ind. Eng.Chem..

[49] Sun YG, Xia YN (2003). Biological applications of localized surface plasmonic phenomena. Proc. SPIE-Int. Soc. Opt. Eng..

[50] Hutter E, Fendler JH (2004). Exploitation of localized surface plasmon resonance. Adv. Mater..

[51] Jin R (2001). Photoinduced conversion of silver nanospheres to nanoprisms. Science.

[52] Samanta S, Sarkar P, Pyne S, Sahoo GP, Mishra A (2012). Synthesis of silver nanodiscs and triangular nanoplates in PVP matrix: photophysical study and simulation of UV-vis extinction spectra using DDA method. J. Mol. Liq..

[53] O’Brien MN, Jones MR, Kohlstedt KL, Schatz GC, Mirkin CA (2015). Uniform circular disks with synthetically tailorable diameters: two-dimensional nanoparticles for plasmonics. Nano Lett..

[54] Jin R (2003). Controlling anisotropic nanoparticle growth through plasmon excitation. Nature.

[55] Ghosh T, Bardhan M, Bhattacharya M, Satpati B (2015). Study of inelastic mean free path of metal nanostructures using energy filtered transmission electron microscopy imaging. J. Microsc..

[56] Ameer FS (2016). Tuning localized surface plasmon resonance wavelengths of silver nanoparticles by mechanical deformation. J. Phys. Chem. C.

[57] Senapati D (2011). A label-free gold-nanoparticle-based SERS assay for direct cyanide detection at the parts-per-trillion level. Chem. Eu. J..

[58] Sun Y, Mayers BT, Xia Y (2002). Template-engaged replacement reaction: aone-step approach to the large-scale synthesis of metal nanostructures with hollow interior. Nano Lett..

[59] Metraux GS, Cao YC, Jin R, Mirkin CA (2003). Triangular nanoframes made of gold and silver. Nano Lett..

[60] Ghosh T, Satpati B, Senapati D (2014). Characterization of bimetallic core-shell nanorings synthesized via ascorbic acid-controlled galvanic displacement followed by epitaxial growth. J. Mater. Chem. C..

[61] Borsook H, Keighley G (1993). Oxidation-reduction potential of ascorbic acid (Vitamin C). Proc. Nat. Ac. Sc..

