# Supplementary material for: Decoupled in-plane Dipole Resonance Modulated Colorimetric Assay-Based Optical Ruler for Ultra-Trace Gold (Au) Detection
Source: Sci Rep. 2018 Jan 17;8:868. doi: 10.1038/s41598-018-19148-w (PMC5772471; doi:10.1038/s41598-018-19148-w)
Supplement: Supplementary file 1 — Supplementary Information [file 41598_2018_19148_MOESM1_ESM.pdf]

Decoupled *in-plane* Dipole Resonance Modulated Colorimetric Assay-Based Optical Ruler for Ultra-Trace Gold (Au) Detection

Ajoy Mandal,[†#] Maireyee Bhattacharya,[†#] Denis V. Kuznetsov,[§] Tapas Ghosh,[‡] Sudeshna Das Chakraborty,[†] Biswarup Satpati,[‡] Vsevolod Mazov,[§] Dulal Senapati[†]*

[†]Chemical Sciences Division and [‡]Surface Physics & Material Science Division, Saha Institute of Nuclear Physics, HBNI, 1/AF, Bidhannagar, Kolkata-700064, India.

[§]Department of Functional Nanosystems and High Temperature Materials, National University of Science and Technology 'MISiS', Leninsky, Prospect 4, 119049 Moscow, Russia

[#]Authors have equal contribution

Growth Solution Preparation:

Materials

Silver nitrate ($AgNO_3$; Bioxtra, 99% (titration)), gold (III) chloride trihydrate ($HAuCl_4.3H_2O$; ≥99.9%, trace metals basis), sodium citrate tribasic dihydrate ($C_6H_5Na_3O_7.2H_2O$; BioUltra, for molecular biology, ≥99.5% (NT)) and L-ascorbic acid (BioXtra, ≥99.0%, crystalline) were purchased from Sigma Aldrich and used without any further purification. All the glassware used in the experiment was cleaned by extran liquid detergent and then washed thoroughly with double distilled water and finally with Milli-Q water and dried in an oven before use. For all of the preparation steps we used Milli-Q water with a resistivity of 18.2MΩcm.

Growth solution preparation

Under continuous stirring condition 175µL of 1% sodium citrate tribasic dihydrate (TSC), 250µL of 10^{-2}M silver nitrate ($AgNO_3$) and 50µL of 10^{-1}M of ascorbic acid (AA) were added one-after-another into 10mL of Milli-Q water. We named this homogeneous solution as 'growth solution'.

Synthesis of silver nanoparticles (AgNPs)

Under continuous stirring condition different concentration of Au-ion was added to the 'growth solution' and the whole solution turned into colored solution within few minutes, indicating the formation of silver nanoparticles (AgNPs). The reaction was carried out at room temperature in a 15mL Borosil glass bottle placed on a magnetic stirrer allowing continuous mixing of chemicals. The reaction was allowed to proceed for another ~2min after the addition of all reagents simultaneously. We also added different metal ion to the solution to see their influence in AgNP formation. The schematic representation of the stepwise synthetic procedure of the Au-ion catalyzed AgNP synthesis has been explained in **Figure 1(I)**.

Characterization:

Spectroscopic Characterization

The absorption spectra of dispersed AgNPs were measured with an UV–vis spectrophotometer Jasco V650 at room temperature (25ºC). Details about their plasmon peak appearance due to the production of Au-ion catalyzed AgNDs along with spherical-AgNPs have been described in details in the main text. Associated controlled experiments to confirm the necessity of Au-ion's presence has been tested in a systematic way. Influence of other heavy metals, precious metals and alkali metals for the possible false positive sensing or for the improvement of the sensitivity of gold ion detection has also been discussed in details in the main text.

Confirmations of AgNDs formation, their size variation, and crystallographic information of the generated silver nanoparticles in presence of different concentrations of Au-ions have been done by TEM characterization. We have used simple but modified techniques for clean monolayer sample preparation for TEM measurement. We used 300 mesh copper formvar/carbon grid throughout the measurement. A dip-and-dry technique has been adopted to make TEM samples. After completion of the reaction, a TEM grid was immersed in the concentrated nanomaterial sample solution using tweezers, and the hydrophobic carbon coating allowed the formed monolayer of the sample to stick onto the copper mesh, which was dried on a soft tissue paper. After complete drying, the resulting grid was used for TEM. Details of the electron microscopy have been described in the Experimental Methods section of main text. For High Resolution

Transmission Electron Microscopic (HRTEM) measurements we used a FEI, Tecnai G^2F30, S-Twin microscope operating at 300kV. The compositional analysis was performed by energy dispersive X-ray spectroscopy (EDS, EDAX Instruments) attachment on the Tecnai F30.

Out-of-Plane Quadrupole Resonance:

Due to the very low intensity and presence of several absorbance profiles, the *out-of-plane* quadrupole resonance near 335nm is not clearly visible in **Figure 1(II)** of the main text and plotted separately as **Figure 1S**. This clearly shows a hump at 336nm along with two intense plasmon band, one at 400nm due to the *out-of-plane* dipole resonance (transverse plasmon band) and another one at 525nm due to the *in-plane* dipole resonance (longitudinal Plasmon band).

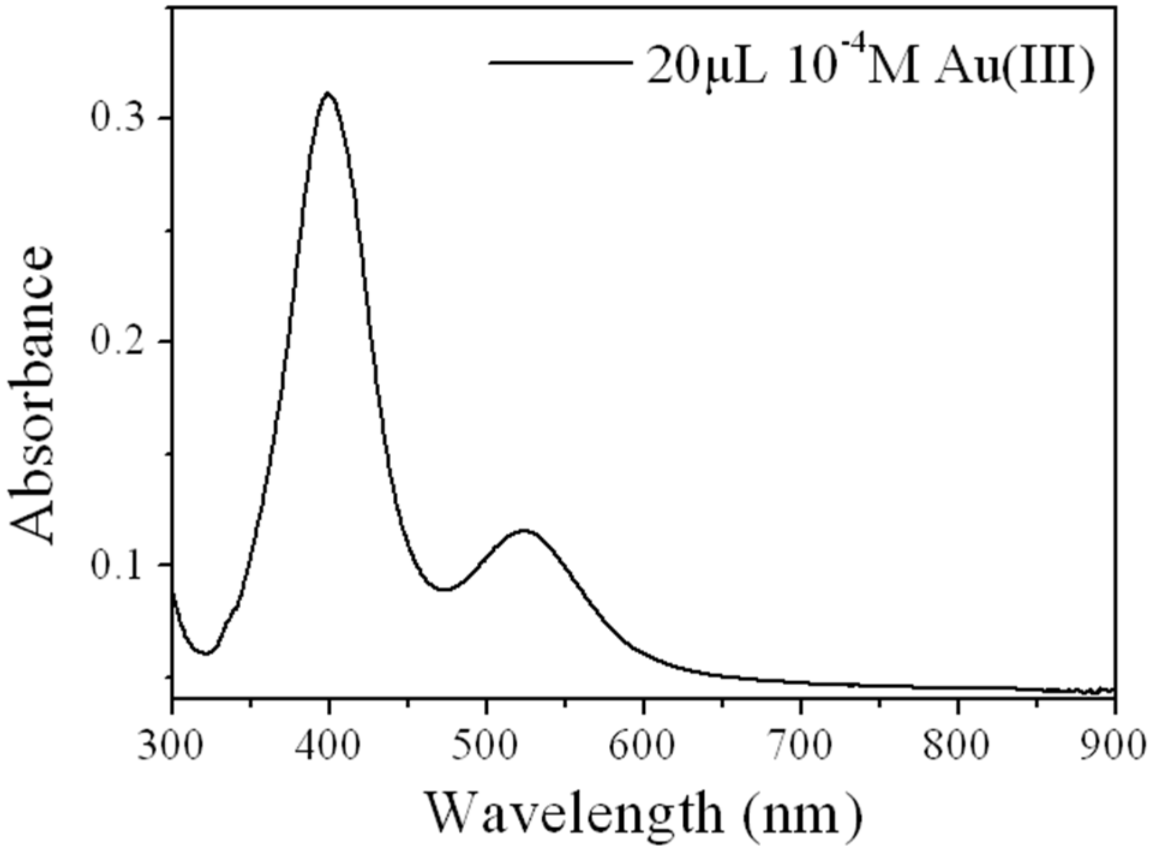

Figure 1S: *Out-of-plane* quadrupole resonance at 336nm observable from the 'growth solution' with 40*ppb* Au-ion concentration.

Kinetics of the Growth of the AgNDs:

Stability of the produced AgNDs and their real time structural change at different concentration of Au-ions has also been studied by performing real-time UV-vis spectroscopy. It is clearly observable from **Figure 2S** that the *in-plane* dipole resonance peak or the longitudinal plasmon band (685.5nm for Set-E and 770nm for Set-F of **Figure 1(I)**) shifts gradually towards shorter wavelength. This gradual blue shifting of the coupled plasmon peak correlates to the formation of AgNDs of smaller particle size and hence the reduced aspect ratios as the particles get stabilized. Kinetics of morphology transition for two different sets (Set-E with 10*ppb* Au-ion and Set-F with 6*ppb* Au-ion) has been explained in **Figure 2S**.

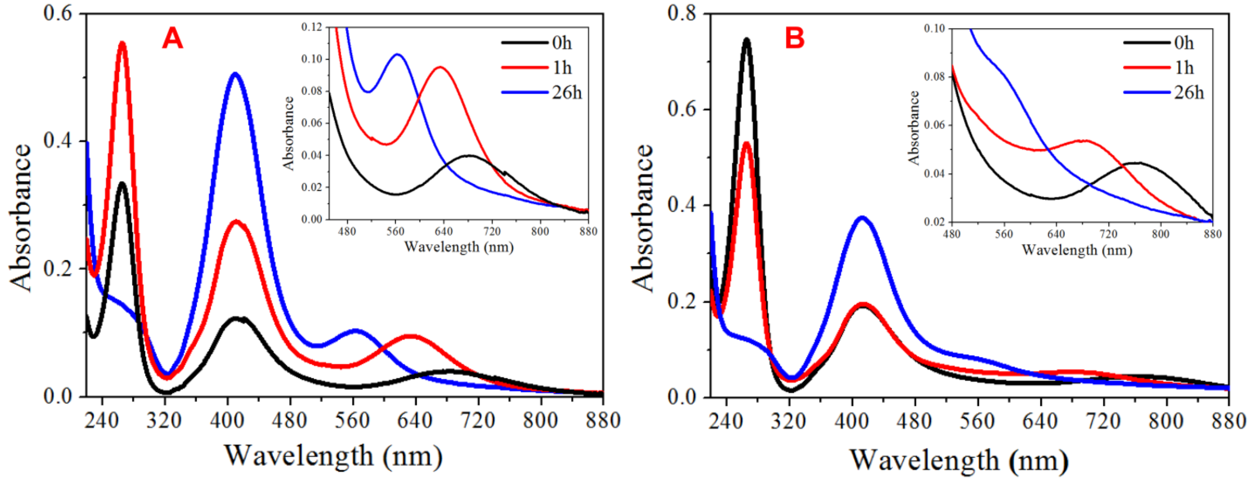

Figure 2S: Kinetics of morphology transition for two different sets, (A) Set-E with 10*ppb* Au-ion and (B) Set-F with 6*ppb* Au-ion. Kinetics study has been performed over 26 hours for both the samples, keeping all the other physical parameters constant except the concentration of Au-ion.

Analytical Method for Calculating Relative Stress for a Disc and a Sphere at Same Optical Force:

The extent of plasmon shifting for a nanodisc to that of a nanosphere by applying same optical force can be calculated analytically. The aspect ratio for any sphere, AR_{sphere}, is always 1.

However, that of a nanodisc of diameter d and of thickness h is d/h, so AR_{disc} increases as d increases for a fixed value of h. Let us consider five nanospherers: S1, S2, S3, S4, S5 and five nanodiscs: D1, D2, D3, D4, D5 (**Figure 3SA**). By considering five spheres and five discs with diameters, d = 10nm, 20nm, 30nm, 40nm, 50nm and a constant thickness of all the nanodiscs as 9.75nm (relative thickness of nanodisc is about 0.065 which corresponds to about 9.75nm (by considering λ at 150nm), the average thickness of the nanodisc), calculated AR_{disc} are presented in **Table 3SB**.

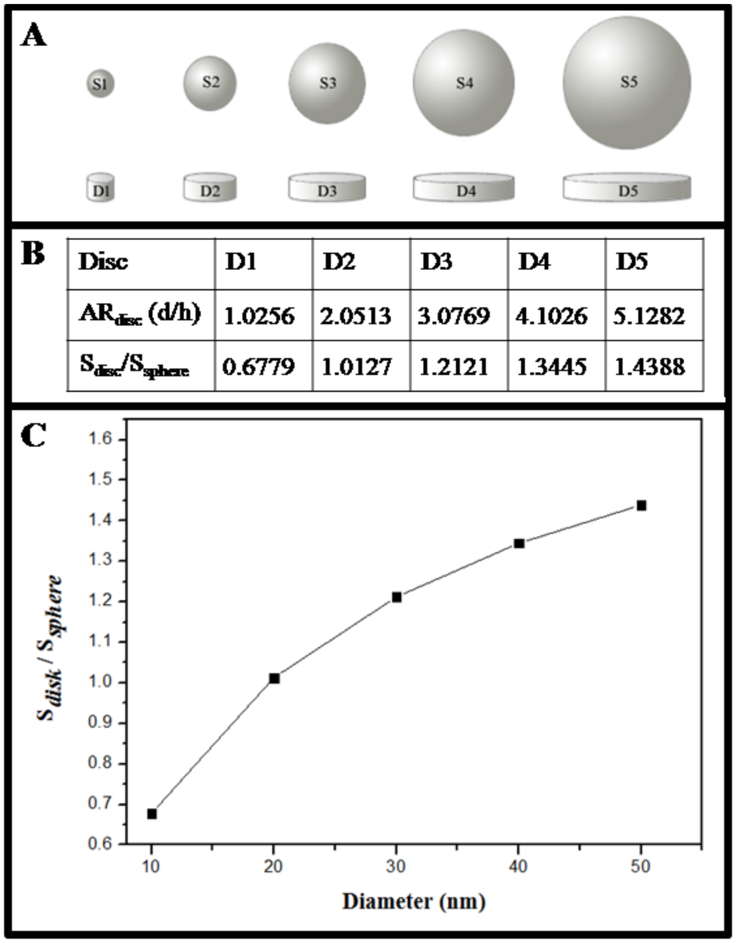

Disc	D1	D2	D3	D4	D5
AR_{disc} (d/h)	1.0256	2.0513	3.0769	4.1026	5.1282
S_{disc}/S_{sphere}	0.6779	1.0127	1.2121	1.3445	1.4388

Figure 3S. (A) Variation of aspect ratio of nanodisc by varying their diameter (d) at a fixed thickness (h). Aspect ratio of a sphere with any diameter is always 1, (B) Gradual increment of the ratio of stress between disc and sphere at a particular optical force as the aspect ratio of nanodisc increases by varying their diameter (d) at a fixed thickness (h) and (C) Variation of relative stress between disc and sphere with disc diameter at a constant disc thickness.

The extent of plasmon shift for a nanostructure towards higher wavelength may be correlated to the easiness with which the electron cloud over it can take part in collective oscillation. The more accessible and pliant the electron cloud is the less is the energy required to excite them. Gradual red shifting of plasmon is expected if the amount of required excitation energy also reduces gradually. Now, this pliancy is a measure of how easily the electron cloud can be disturbed or deformed.

Theoretically, we can have an idea of this by calculating the stress factor.

$$Stress(S) = \frac{Force(F)}{Area\ (A)}$$

If r = radius of the disc = radius of the sphere and h= thickness of the disc, then

Surface area for the disc, $A_{disc} = 2\pi r^2 + 2\pi rh = 2\pi r(r + h)$

and Surface area for the sphere, $A_{sphere} = 4\pi r^2$

The ratio of stress between disc and sphere at a particular optical force is then defined as

$$S_{disc}/S_{sphere} = A_{sphere}/A_{disc} = 4\pi r^2/2\pi r(r + h) = 2r/(r + h) = \left(2r/h\right)\Big/\left\{(2r + 2h)/2h\right\}$$

$$= AR_{disc}\Big/\left\{\left(AR_{disc}/2\right) + 1\right\}$$

Where AR_{disc} = Aspect Ratio of the Disc = d/h = 2r/h

By applying the above formula, values of S_{disc}/S_{sphere} are obtained for the discs which have been provided in **Table 3SB** and the corresponding plot of S_{disc}/S_{sphere} vs. Diameter has been presented in **Figure 3SC**. From **Figure 3SC** it is clearly evident that S_{disc}/S_{sphere} increases with

increasing the diameter which means stress is more effective in case of a nanodisc than a nanosphere but the extent of this increase of stress reduces gradually as higher value of diameter is approached. This increment of stress with diameter of nanodisc can be compensated by relaxing the electron cloud on nanosurface more easily and results surface plasmon shifting into red wing as we observed from the recorded tunable plasmon spectra (**Figure 1(II)**). Stress induced tuning of localized surface plasmon resonance wavelength for silver nanoparticle by mechanical force has been reported before by J. N. Aner *et al.*[56] In this work we have reported optical force induced large scale plasmon tuning simply by changing their aspect ratio where the induced stress directly depends on their aspect ratios.

Controlled Experiments:

Several controlled experiments have been performed to make sure that we need the pre formulated ‘growth solution’ to achieve the color coded sensing of Au-ion. **Figure 4S(I) and (II)** clearly explain the necessity of the ‘growth solution’ composition to generate *in situ* AgNDs to generate different colors in presence of variable amount of soluble gold impurity. We have performed a number of controlled experiments to test the necessity of all of the four (4) components namely, TSC, Ag-ion, AA, and Au-ion in the ‘growth solution’, which are as follows:

(i) We performed the same experiment in absence of TSC, which known to be a stabilizing agent for spherical gold and silver nanoparticle synthesis, cannot generate any nanoparticle (gold or silver nanoparticle) and hence we have not observed any color appearance. This is clearly observable from the color of left most bottle of **Figure 4S(I)**. This directly proves that the adequate amount of TSC is an essential component of the ‘growth solution’ to perform the color coded sensing of Au.

(ii) Next we explored the role of AA by performing the colorimetric test in absence of AA while keeping all the other components present in the solution. In absence of Ascorbic acid, which generally acts as reducing agent (can act also as a stabilizing agent[1]), we could not achieve

the reduction of Ag^+ and the subsequent formation of AgNDs and resultant color coding. The essential role of AA in the 'growth solution' is clearly visible from the color of the second bottle.

(iii) In the third bottle where we have added neither TSC nor AA and as we expect, addition of Au-ion does not produce any AgNDs and hence doesn't show any colorimetric change.

(iv) To make sure that the appearance of color in presence of soluble Au-ion is not due to the reduction of gold ions by AA, we performed the experiment in absence of TSC and $AgNO_3$. Generation of no color indicates that the added amount of Au-ion is not sufficient to show any visible color arising from gold nanoparticles which has been demonstrated in fourth bottle of **Figure 4S(I)**.

(v) Bottle 5 represents the ideal case when all the essential components of the 'growth solution' are present in adequate amount and shows the yellow color from the generated AgNDs due to the addition of 500*ppb* Au(III) in the 'growth solution'.

(vi) For further confirmation of the fact that the colours are originating from Ag nanoparticles and not interfered by any Au nanoparticle, we performed another set of controlled experiments by varying the gold solution concentration from 2*ppb* to 1000*ppb* (bottle A-J) in absence of silver nitrate solution which showed no noticeable colour arising in the visible region as reflected in **Figure 4S(II)**.

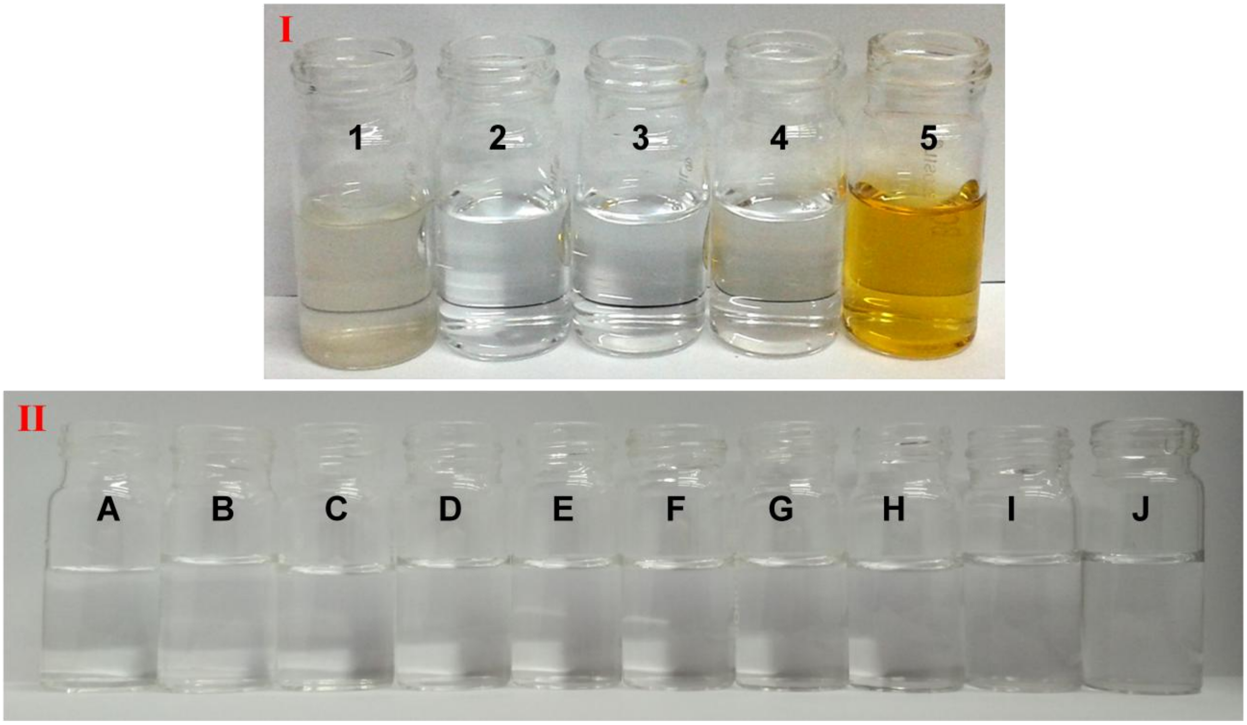

Figure 4S: (I) 10mL water solution containing (1) 250µL 10^{-2}M $AgNO_3$ + 5µL 10^{-4}M $HAuCl_4$ + 50µL 100mM AA, (2) 175µL 1% TSC+ 250µL 10^{-2}M $AgNO_3$ + 5µL 10^{-4}M $HAuCl_4$, (3) 250µL 10^{-2}M $AgNO_3$ + 5µL 10^{-4}M $HAuCl_4$, (4) 5µL 10^{-4}M $HAuCl_4$ + 50µL 100mM AA, (5) 175µL 1% TSC + 250µL 10^{-2}M $AgNO_3$ + 50µL 100mM AA + 2.5µL 10^{-2}M (or 250µL 10^{-4}M) $HAuCl_4$ or 500*ppb* $HAuCl_4$. (II) 10mL water solution containing 175µL 1% TSC + 50µL 100 mM AA + variable amount 10^{-4}M $HAuCl_4$ (A – 1µL or 2*ppb*, B – 3µL or 6*ppb*, C – 5µL or 10*ppb*, D – 10µL or 20*ppb*, E – 20µL or 40*ppb*, F – 50µL or 100*ppb*, G – 100µL or 200*ppb*, H – 200µL or 400*ppb*, I – 350µL or 700*ppb*, J – 500µL or 1000*ppb*).

The effect of higher concentration of Au-ion (>200*ppb*) on the 'growth solution' has been shown in **Figure 5S**. At higher concentration of Au-ion, 'growth solution' predominantly generates gold nanoparticles and subsequently colour varies from yellow to red and finally to brown (**Figure 5SA**) with a strong plasmon near 500nm which is the characteristic plasmon peak for spherical gold nanoparticles (**Figure 5SB**). **Figure 5S** also explains that the presence of $HgCl_2$ which enhances the detection sensitivity of gold by three (3) times, alone can't produce any color change; rather produce a gray color originating from insoluble AgCl.

At lower concentration of Au-ion (up to 1μM), plasmon band is predominated by silver nanoparticle with characteristic plasmon band at 400nm. As we increase the concentration of Au-ion, plasmon band shifts to higher wavelength near 500nm.

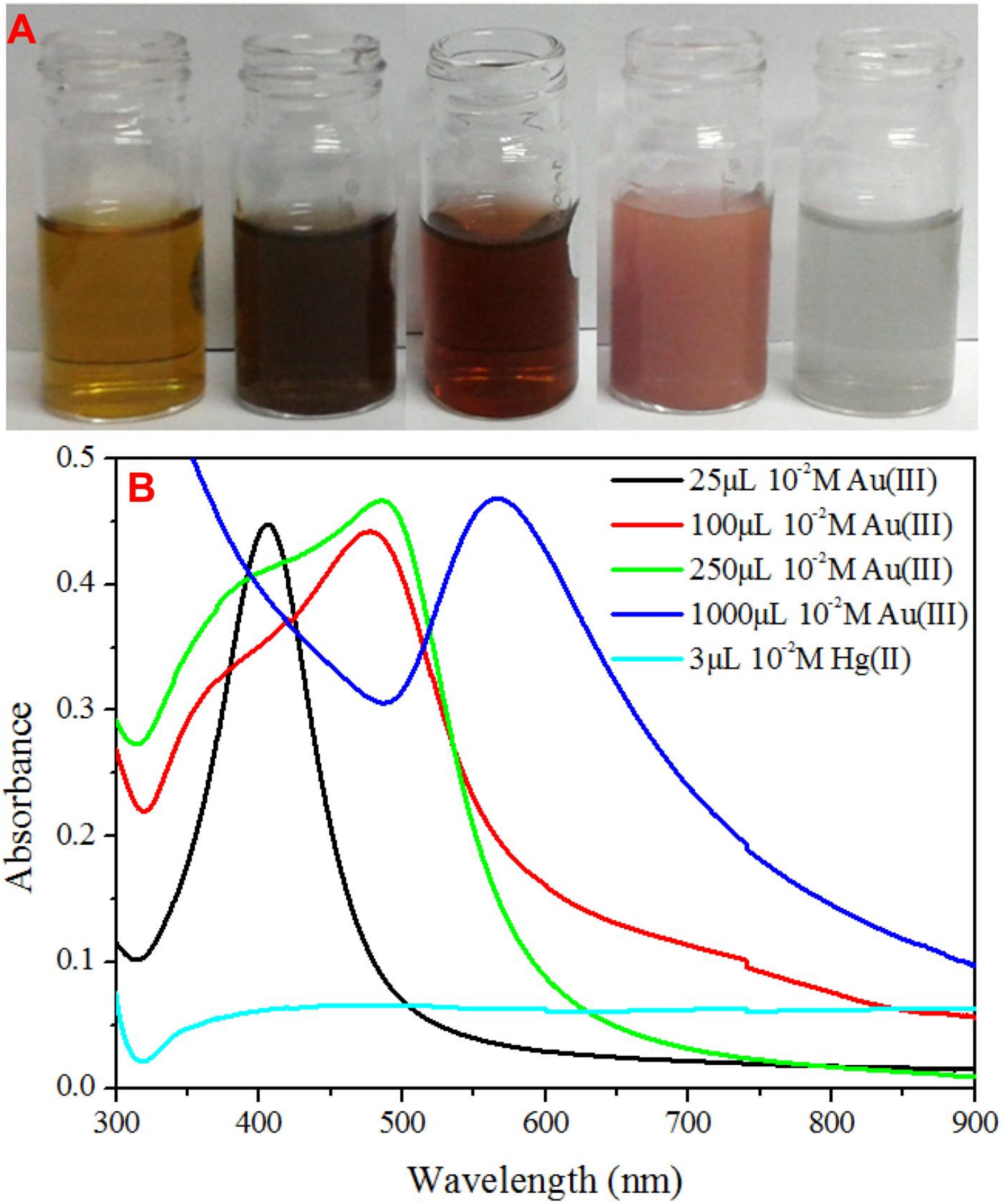

Figure 5S. (A) (Left to right): 10mL water with 175µL 1% TSC, 250µL 10^{-2}M $AgNO_3$, 50µL 100mM AA, and different amount of 10^{-2}M soluble gold (1) 25µL, (2) 100µL, (3) 250µL, (4) 1000µL, and (5) 3µL of 10^{-2}M Hg^{2+} in absence of Au-ion. (B): Plasmonic response of 'growth solution' as we increase the concentration of Au-ion from ~1µM to 1mM.

Composition Analysis of Synthesized AgNDs:

Elemental mapping of synthesized AgNDs have been performed by recording their EDX spectra. EDX spectra rely on the interaction between incident electrons and the materials inside the nanoparticles to provide unique set of X-ray emission[2] which gives the signature of the elemental composition[2]. Our measured EDX spectra in **Figure 6S** show trace of Au only when the added Au-ion concentration is above 40*ppb*. Below 40*ppb* of Au-ion, recorded EDX spectra does not show any signature of the Au-ion's presence but could easily be detectable by our colorimetric assay-based optical ruler which indirectly shows the sensitivity of our 'growth solution' based methodology for trace level of Au-ion detection.

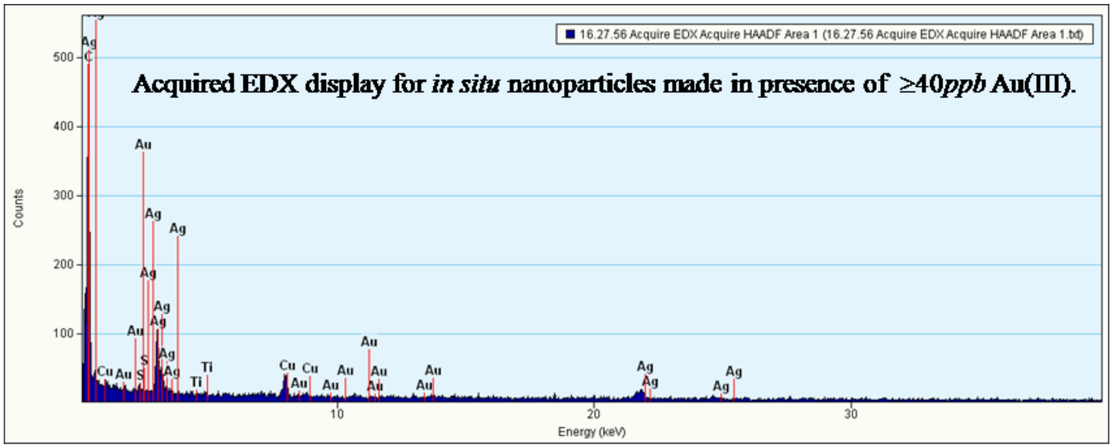

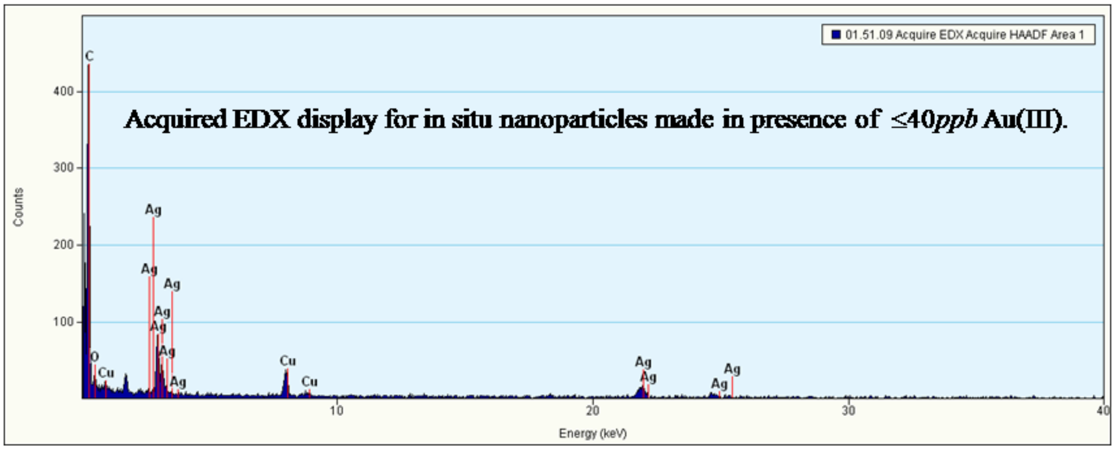

Figure 6S: EDX spectra of the *in situ* generated AgNDs from the 'growth solution' in presence of Au-ion (top) ≥ 40*ppb* and (bottom) ≤ 40*ppb*.

Role of Au-ion as Catalyst:

Striking role of Au-ion tracer has been explained in details in the main text. Though the differential reduction potential between Ag^{+}/Ag^{0} and AA/AA^{2-} which is ~0.8V results a very low tendency to reduce Ag^{+} to Ag^{0} for AgNP formation and a large portion of AA (absorbance at 265.5nm) remain unreacted, presence of substantial amount of Au-ions changes the situation dramatically as the reduction potential of Au^{3+}/Au^{0} is substantially higher than Ag^{+}/Ag^{0} and the resultant differential reduction potential (between Au^{3+}/Au^{0} and AA/AA^{2-} is 1.5V) forces AA to acts as an efficient reducing agent. As a result, all the available AA is being used in the reduction process which corresponds to the disappearance of 265.5nm peak from UV-vis spectra in **Figure**

7S. Efficient use of AA as a reducing agent, catalyzed and regulated by Au-ion to reduce the available metal ions present in the 'growth solution' is clearly observable from **Figure 7S.**

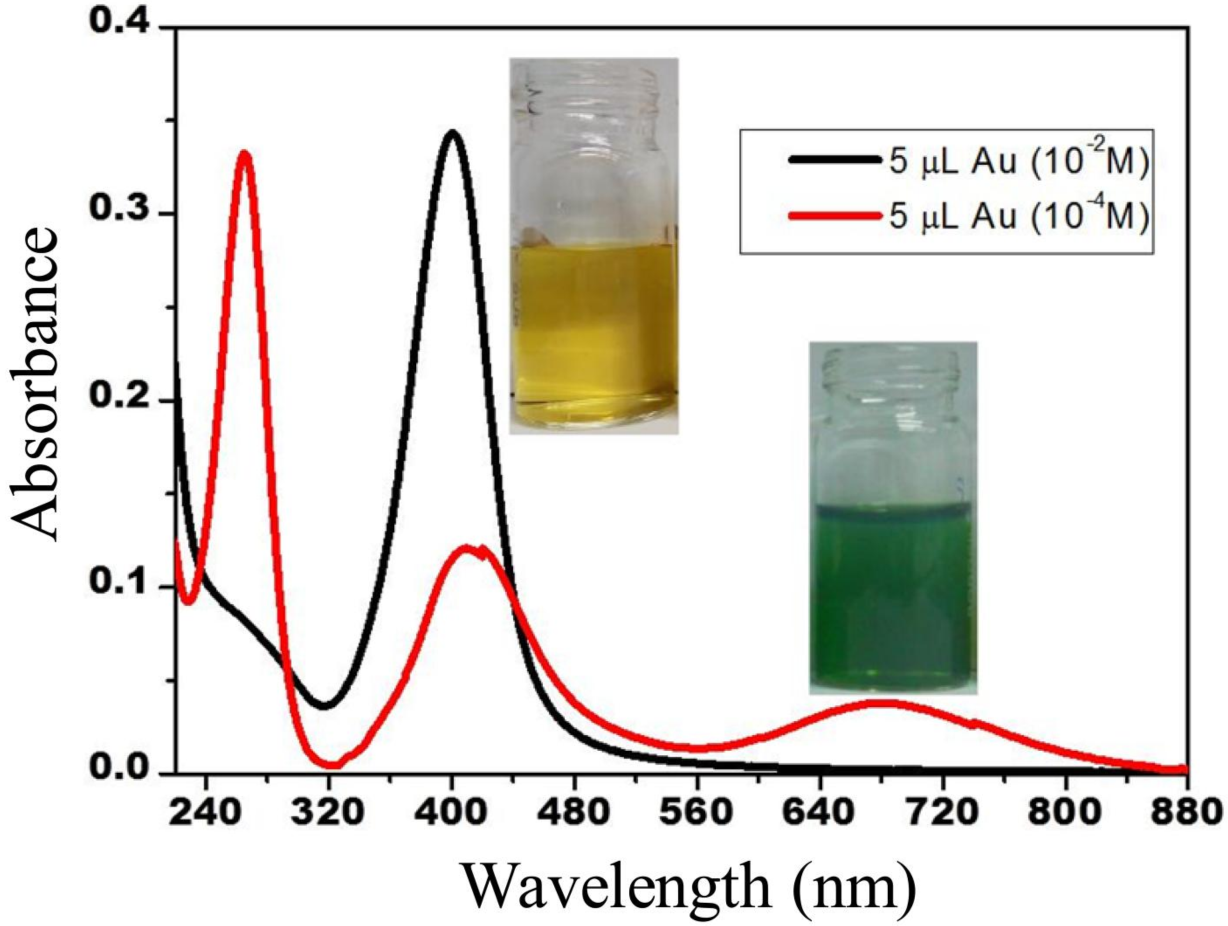

Figure 7S: Au-ion regulated differential reduction potential to efficiently use AA as reducing agent. Disappearance of 265.5nm absorbance originating from AA proves the efficient use of AA as a component in the 'growth solution' to reduce Ag-ion in presence of substantial amount of Au-ion.

Operating Parameters of the ICP-OES:

We cross examined the quantity of Au-ions obtained from our turn-on colorimetric assay and optical ruler by ICP-OES to measure the actual Au-ion concentration. A 1000*ppm* Au solution from NIST was used as standard for ICP-OES calibration. Details of the operating conditions employed are provided in **Table 1S**.

Table 1S: The operating parameters of the ICP-OES used for the measurement of Au-ion tracer

Method parameters	
RF power (W)	1150
Nebulizer flow (L/min)	0.5
Auxiliary flow (L/min)	1
Coolant gas flow (L/min)	12
Sample chamber	Cyclonic
Pump rate (rpm)	50
Replicates	3
Standard rinse time (s)	5
Plasma view	Radial

Element	Emission line (nm)
Au	201.2
	208.2
	242.5
	267.5

Calculation of Average Error

We have taken 5, 10, 25, 50 and 90μL from the stock solution with unknown Au-ion concentration and experimentally measured their concentration by using different analytical methods (ICP-OES, Optical and Colorimetric techniques). Obtained results have been tabulated in Table 2S for these five different samples (A: 5μL, B: 10μL, C: 25μL, D: 50μL and E: 90μL of unknown gold solutions respectively). In our study the % of error both for optical and colorimetric assay compared to the standard ICP-OES assay has been calculated by considering the following equations:

$$\% \text{ Error}_{\text{optical}} = \left| \frac{[Au]_{optical} - [Au]_{ICP-OES}}{[Au]_{ICP-OES}} \right| \times 100 \quad (1)$$

$$\% \text{ Error}_{colorimetric} = \left| \frac{[Au]_{colorimetric} - [Au]_{ICP-OES}}{[Au]_{ICP-OES}} \right| \times 100 \quad (2)$$

By considering these equations, obtained error table both for optical and colorimetric assay is as follows:

Table 2S: Au-ion concentration measurement from ICP-OES, colorimetric and optical assay and the % of error calculated for colorimetric and optical assay with respect to the standard ICP-OES method.

Sample	A	B	C	D	E
$[Au]_{ICP-OES}$ (ppb)	7.365	10.8	18.392	43.7	110.61
$[Au]_{optical}$ (ppb)	4	10	23	37	100
$[Au]_{colorimetric}$ (ppb)	6	10	20	40	100
% $\text{Error}_{optical}$	45.689	7.407	25.054	15.331	9.592
% $\text{Error}_{colorimetric}$	18.533	7.407	8.742	8.466	9.592

By considering all the data points the obtained average error for optical and colorimetric assays are given below:

Average $\text{Error}_{optical}$ = 20.6146%

Average $\text{Error}_{colorimetric}$ = 10.548%

$$\text{Average Error} = \frac{Average\ \ Error_{\ optical}\ + Average\ \ Error_{\ colorimetric}}{2} = 15.5813\% \approx 16\%$$

It is clearly evident from the % of error table that the error point for optical assay at the lowest gold concentration (**Sample A**) is close to 50% and hence we can consider this data point as an

outlier (In statistics, an outlier is an observation point that is distant from other observations) data point to remove from the list to calculate average % of error. By considering so, the obtained average error for optical and colorimetric assays is as follows:

Average $Error_{optical}$ = 14.346%

Average $Error_{colorimetric}$ = 10.548%

Average Error = $\frac{Average\ \ Error_{\ optical}\ +Average\ \ Error_{\ colorimetric}}{2} = 12.447\% \approx 12\%$

References:

1. Senapati, D., Dasary, S. S. R., Singh, A. K., Senapati, T., Yu, H. & Ray, P. C. A label-free gold-nanoparticle-based SERS assay for direct cyanide detection at the parts-per-trillion level. *Chem. Eu. J.* **17**, 8445-8451 (2011).

2. Goldstein, J. *Scanning Electron Microscopy and X-Ray Microanalysis*, Springer.
